# Development and validation of a nomogram based on CT images and 3D texture analysis for preoperative prediction of the malignant potential in gastrointestinal stromal tumors

**DOI:** 10.1186/s40644-019-0284-7

**Published:** 2020-01-13

**Authors:** Caiyue Ren, Shengping Wang, Shengjian Zhang

**Affiliations:** 1 Department of Nuclear Medicine, Shanghai Proton and Heavy Ion Center, Shanghai, 201315 China; 2 Department of Radiology, Fudan University Shanghai Cancer Center, 270 Dong’ an Road, Shanghai, 200032 China

**Keywords:** Gastrointestinal stromal tumors, Grade, Nomogram, Computed tomography, Texture analysis

## Abstract

**Background:**

Gastrointestinal stromal tumors (GISTs), which are the most common mesenchymal tumors of the digestive system, are treated varyingly according to the malignancy. The purpose of this study is to develop and validate a nomogram for preoperative prediction of the malignant potential in patients with GIST.

**Methods:**

A total of 440 patients with pathologically confirmed GIST after surgery in our hospital from January 2011 to July 2019 were retrospectively analyzed. They were randomly divided into the training set (*n* = 308) and validation set (*n* = 132). CT signs and texture features of each patient were analyzed and predictive model were developed using the least absolute shrinkage and selection operator (lasso) regression. Then a nomogram based on selected parameters was developed. The predictive effectiveness of nomogram was evaluated by the area under receiver operating characteristic (ROC) curve (AUC). Concordance index (C-index) and calibration plots were formulated to evaluate the reliability and accuracy of the nomogram by bootstrapping based on internal (training set) and external (validation set) validity. The clinical application value of the nomogram was determined through the decision curve analysis (DCA).

**Results:**

Totally 156 GIST patients with low-malignant (very low and low risk) and 284 ones with high-malignant potential (intermediate and high risk) are enrolled in this study. The prediction nomogram consisting of size, cystoid variation and meanValue had an excellent discrimination both in training and validation sets (AUCs (95% confidence interval(CI)): 0.935 (0.908, 0.961), 0.933 (0.892, 0.974); C-indices (95% CI): 0.941 (0.912, 0.956), 0.935 (0.901, 0.982); sensitivity: 81.4, 90.6%; specificity: 75.0, 75.7%; accuracy: 88.0, 88.6%, respectively). The calibration curves indicated a good consistency between the actual observation and nomogram prediction for differentiating GIST malignancy. Decision curve analysis demonstrated that the nomogram was clinically useful.

**Conclusion:**

This study presents a prediction nomogram that incorporates the CT signs and texture parameter, which can be conveniently used to facilitate the preoperative individualized prediction of malignancy in GIST patients.

## Background

Gastrointestinal stromal tumors (GISTs) are the most common mesenchymal tumors of the digestive system with malignant potential regardless of its size, accounting for 1–3% of all gastrointestinal tumors [1, 2]. GISTs are classified as very low, low, intermediate and high risk which the malignant potential increases in turn according to the 2008 National Institutes of Health (NIH) criteria [3, 4], which are the main reference standards for prognosis determined by consensus. Preoperative knowledge of risk classification can provide valuable information for evaluating the adequacy of surgical resection and the need for adjuvant treatment [5–7]. Ultrasound or CT-guided needle biopsy of GISTs for immunological analysis as an easy-to-perform method is commonly used in clinical practice [8]. However, biopsy before operation is not recommended for most GIST patients who can be completely resected [9], and a small amount of pathological tissues in some patients with preoperative biopsy indications fails to meet the need for accurate diagnosis [10]. In addition, improper operation might cause tumor rupture and hemorrhage, increasing the risk of tumor dissemination. Thus, it is clinically important and necessary to explore noninvasive, reliable and practical biomarkers for preoperatively predicting the malignant potential in GIST patients.

Computed tomography (CT) is widely recognized as the main imaging method for the diagnosis, characterization and evaluation of curative effect in GIST patients due to its convenient operation, good image quality and moderate price [11]. The signs on CT images, such as location, size of the lesions, as well as the presence of cystic necrosis and distant metastases, are helpful to preliminarily judge the malignancy of GIST [12, 13]. However, these signs are of limited value for further accurate classification at the molecular level. Texture analysis, as a popular quantitative image post-processing technology in recent years, can objectively reflect the potential biological characteristics and heterogeneity of tumors because of its quantitative extraction and analysis of pixel distribution in the lesion area [14, 15]. Recent reports have shown that texture analysis based on CT scan was of certain value for prediction the malignancy in GISTs, which could provide a clinical basis for early diagnosis and treatment [16, 17].

Hence, the aim of this study was to develop and validate a preoperative nomogram, incorporating both the CT signs and texture features, for prediction of the malignant potential in patients with GISTs.

## Methods

### Patients

This study was approved by the ethics committee of our hospital. The requirement for informed consent was waived for this retrospective study. Records for GIST patients attending our hospital from January 2011 to July 2019 were obtained. The inclusion criteria including the following: 1) patients who underwent surgery for GISTs with curative intent; 2) information of postoperative pathologically confirmed GISTs risk category available; 3) standard contrast-enhanced CT less than 30 days before surgery. The exclusion criteria including the following: 1) previous history of GISTs or other cancer; 2) preoperative therapy (radiotherapy, chemotherapy or chemoradiotherapy); 3) poor image quality affects lesion segmentation.

A total of 440 patients with pathologically confirmed GISTs who underwent surgical resection were enrolled: 233 males and 207 females; mean age, 58.4 ± 10.87 years; range, 29–87 years. Patients were divided into training set (*n* = 308, 164 males and 144 females; mean age, 57.8 ± 10.54 years; range, 29–87 years) and validation set (*n* = 132, 69 males and 63 females; mean age, 58.5 ± 11.31 years; range, 33–84 years) after simple randomization at a ratio of 7 to 3.

Baseline data pertaining to demographics of each patient, including gender, age, symptom, tumor history, family history was reviewed and recorded.

### Pathological characteristics

All lesions were evaluated for histological characteristics and the expression of CD117 and CD34. The tumors were stratified to very low, low, intermediate and high risk determining by the tumor size, location and mitotic count [3] (Table 1). According to risk categories, the patients in this study were divided into the low-malignant (very low and low risk) and high-malignant (intermediate and high risk) potential group.

**Table 1:** GISTs risk classification of NIH (2008)

Risk category	Tumor size (cm)	Mitotic index (per 50 HPFs)	Primary tumor site
Very low risk	< 2.0	≤5	Any
Low risk	2.1–5.0	≤5	Any
Intermediate risk	2.1–5.0	> 5	Gastric
	< 5.0	6–10	Any
	5.1–10.0	≤5	Gastric
High risk	Any	Any	Tumor rupture
	> 10	Any	Any
	Any	> 10	Any
	> 5	> 5	Any
	2.1–5.0	> 5	Nongastric
	5.1–10.0	≤5	Nongastric

Note: *NIH* National Institutes of Health

### CT image acquisition and analysis

All patients generally underwent contrast-enhanced CT scans on the 32- or 64-slice Siemens Sensation system (Siemens Medical System, Forchheim, Germany). Patients were regularly fasted for 4 to 6 h before the CT examination and encouraged to drink 500–800 mL of water 30 min before the scan and 1000 mL immediately before the scan to fill the gastrointestinal tract. The CT parameters were as follows and used with a standard reconstruction algorithm: tube voltage, 120 kV; tube current, 250–300 mA; slice thickness and interval, 1.5 mm. Patients were in a supine position, and the scan range included all lesion areas. After the unenhanced CT, a total of 80–120 mL (1.5 mL/kg) of iodinated contrast material (Ultravist 370, Bayer Schering Pharma, Berlin, Germany) was injected with a pump injector (Ulrich CT Plus 150, Ulrich Medical, Ulm, Germany) at a flow rate of 3 mL/s into the antecubital vein. The arterial-phase, portal venous-phase and delayed-phase scans were performed at 25 to 30 s, 50 to 60 s and 120 s after the injection of the contrast medium, respectively. The images were uploaded to picture archiving and communication system (PACS) (Carestream, Ontario, Canada) workstations.

Two radiologists with 3 (reader 1) and 13 (reader 2) years of diagnosis experience reviewed and assessed the following 8 CT signs of each lesion without knowing the pathology determined by consensus: the size (the maximal diameter on the largest cross section of tumor), location (gastric and non-gastric), growth pattern (inter-intestinal, extra-intestinal or cross-intestinal), shape (regular or irregular), boundary (clear or unclear) of lesions, and the presence of calcification, cystic necrosis and metastasis.

### Texture feature extraction

**Fig. 1:**
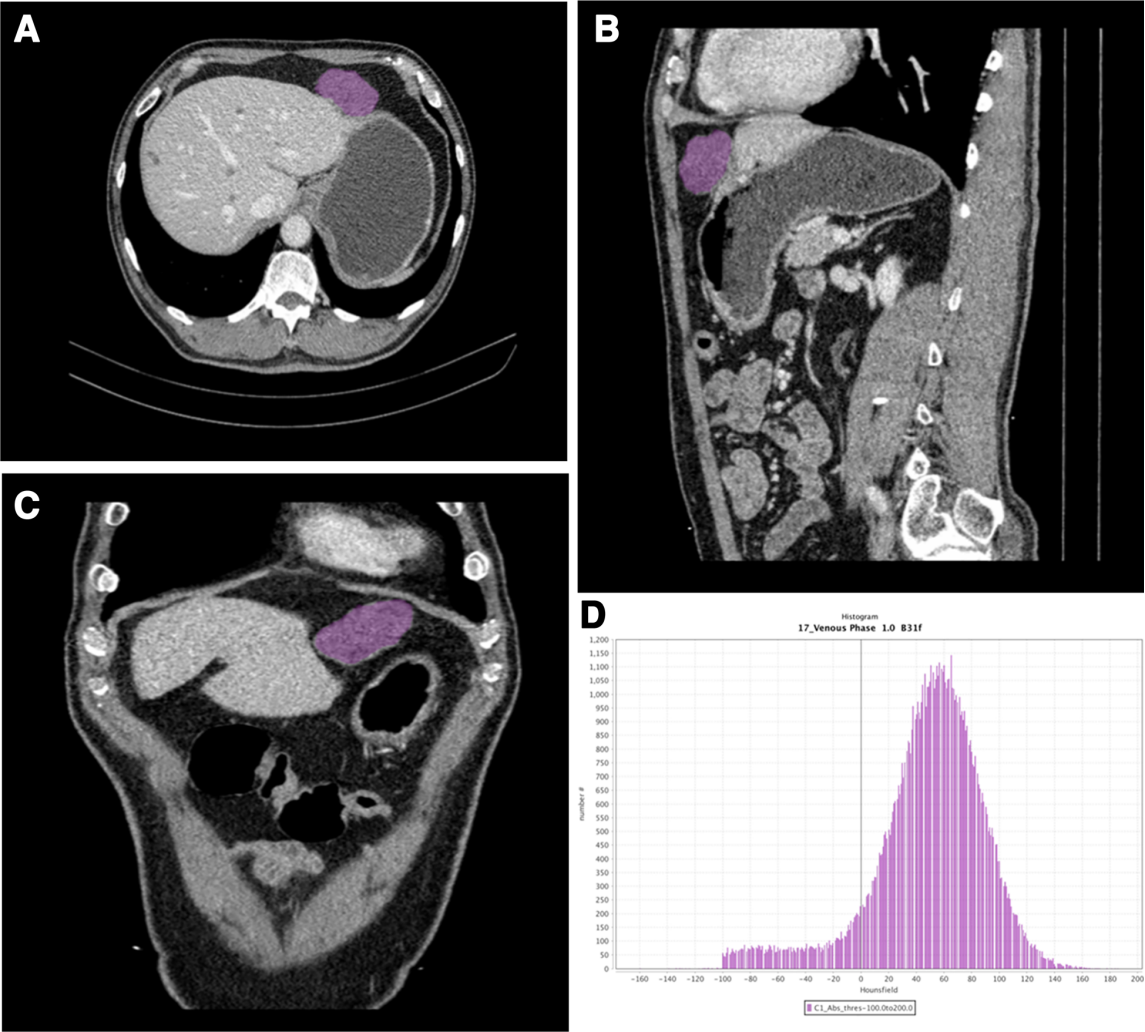
Abdominal portal venous phase CT images of a 33-years-old woman. Texture features were extracted from the primary tumor area (purple overlay). **a** transverse section, **b** median sagittal section, **c** coronal section, **d** Histogram

Feature extraction was performed using LIFEx software (version 4.90; www.lifexsoft.org) with portal venous-phase CT images. The above two radiologists (reader 1, 2) selected the largest slice of the tumor at three-dimensional (3D) images to delineate the region of interest (ROI) by consensus (Fig. 1). The ROI selection should include all tumors and avoid blood vessels, calcification and gas. Intra- and interclass correlation coefficients (ICCs) were used to evaluate the consistency and reproducibility of the intra- and inter-observer agreement of the texture features extraction. An ICC greater than 0.75 indicated good consistency.

Totally 16 three-dimensional texture features were extracted automatically including 9 histogram parameters and 7 Gy-level co-occurrence matrix (GLCM) parameters. A list of the corresponding features is provided in Additional file 1: Table S1, while a detailed description of all features can be found in a study by *Orlhac* [18].

### Statistical analysis

Statistical analysis was performed on R software (version 3.60; http://www.r-project.org). Independent *t-*tests or Mann-Whitney U tests were applied for continuous variables across the groups, and Fisher’s exact tests or χ^2^ tests were used to assess differences in patient categorical variables. A two-sided *P* value of < 0.05 was used as the criterion to indicate a statistically significant difference.

### Establishment of the predictive model and nomogram

Univariate analysis was applied to the clinical demographic parameters, CT signs and texture features to identify the most relevant predictors of the malignant potential of GISTs using Pearson’s correlation test in the training set. Multivariate analysis was performed by lasso (least absolute shrinkage and selection operator) regression with 10-folds cross validation which was used to select the most useful features in previous studies [19, 20] to address multiple cross-related covariates and reduce the risk of overfitting of the data. The prediction model which performed to differentiating low-malignant from high-malignant GIST was developed by the linear fusion of selected features weighted by their coefficients, with a prediction score (Pre-score) calculated for each patient. To provide a quantitative tool to predict malignant potential for GIST patients, we develop a nomogram on the basis of multivariate analysis on training set.

### Predictive performance and validation of nomogram

The prediction performance of nomogram was evaluated by the area under the receiver operating characteristic (ROC) curve (AUC) on both the training and validation sets, with the AUC, sensitivity, specificity and accuracy with 95% confidence intervals (95% CIs) were calculated. Calibration curve was plotted to assess the calibration of the nomogram with the Hosmer-Lemeshow goodness-of-fit test. *P* > 0.05 indicated insignificant deviance from the theoretical perfect calibration. Concordance index (C-index) was formulated to evaluate the reliability and accuracy of the nomogram by bootstrapping (1000 bootstrap resamples) based on internal (training set) and external (validation set) validity.

### Clinical utility of nomogram

The clinical application value of the nomogram was determined through the decision curve analysis (DCA) by quantifying the net benefit to the patient under different threshold probabilities.

## Results

### Clinical and demographic characteristics

Totally 440 GIST patients comprising of 156 low-malignant and 284 high-malignant potential are enrolled in this study. Clinical and demographic characteristics in the training and validation sets are summarized and compared in Table 2. There are no significant differences in gender, age, symptom, tumor history, family history of tumor between the low-malignant and high-malignant potential groups according to the univariate analysis (*p* > 0.05) in either the training or validation sets, consistent with the report [21].

**Table 2:** Clinical and demographic characteristics of patients in the training and validation sets

Characteristics	Training set		*P*-value	Validation set		*P*-value
	Low-malignant group (*n* = 109)	High-malignant group (*n* = 199)		Low-malignant group (*n* = 47)	High-malignant group (*n* = 85)	
Gender			0.641			0.380
Male	60	104		27	42	
Female	49	95		20	43	
Age (mean ± SD, years)	56.9 ± 9.99	58.4 ± 10.82	0.251	57.96 ± 11.20	58.8 ± 11.42	0.696
Symptom (%)			0.875			0.704
+	70 (64.22%)	126 (63.32%)		43 (91.49%)	76 (89.41%)	
-	39 (35.78%)	73 (36.68%)		4 (8.51%)	9 (10.59%)	
Tumor history (%)			0.918			0.343
+	13 (11.93%)	20 (10.05%)		4 (8.51%)	11 (12.94%)	
-	96 (88.03%)	199 (89.95%)		43 (91.49%)	74 (87.06%)	
Family history (%)			0.176			0.187
+	11 (10.09%)	27 (13.57%)		3 (6.38%)	10 (11.76%)	
-	98 (89.91%)	172 (86.43%)		44 (93.62%)	75 (88.24%)	

Note: *P*-values were the results of univariable association analyses of each characteristic and of the two groups

*SD* standard deviation

### Establishment of the predictive model and nomogram

A total of 8 CT signs and 16 texture features were extracted from 440 GIST patients’ CT portal-phase images, and the agreement between the two radiologists (readers 1, 2) was excellent for texture features (all ICCs > 0.85, *p* < 0.05). Thus, the mean measurement values of the two radiologists were used for further analysis.

**Fig. 2:**
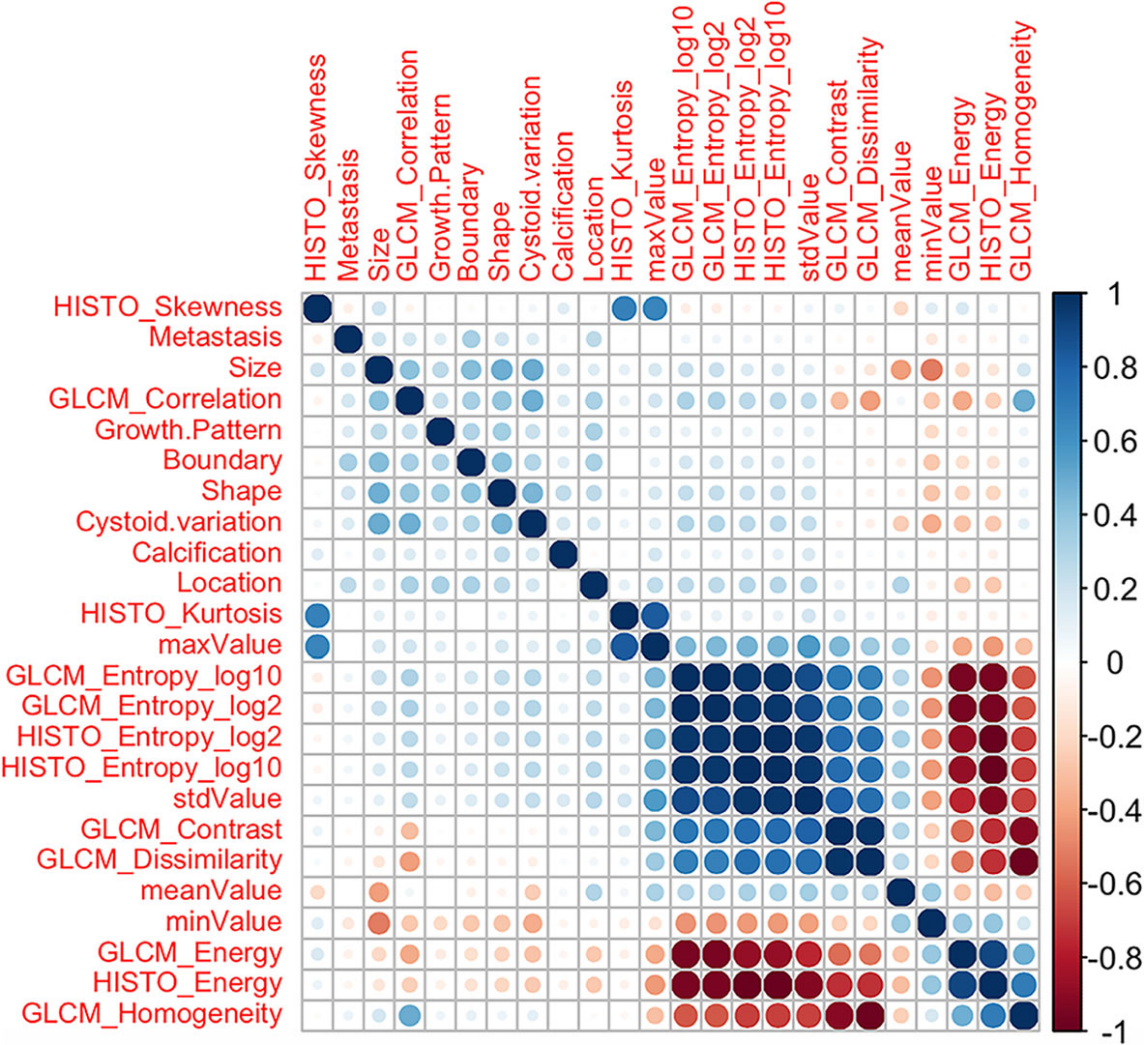
The cross-correlation matrix for covariates used to establish predictive model. The depth of color indicates the intensity of the correlation between covariates. The darker the color, the higher the correlation is. The lighter the color, the lower the correlation is. Blue represents positive correlation and red represents negative correlation

**Fig. 3:**
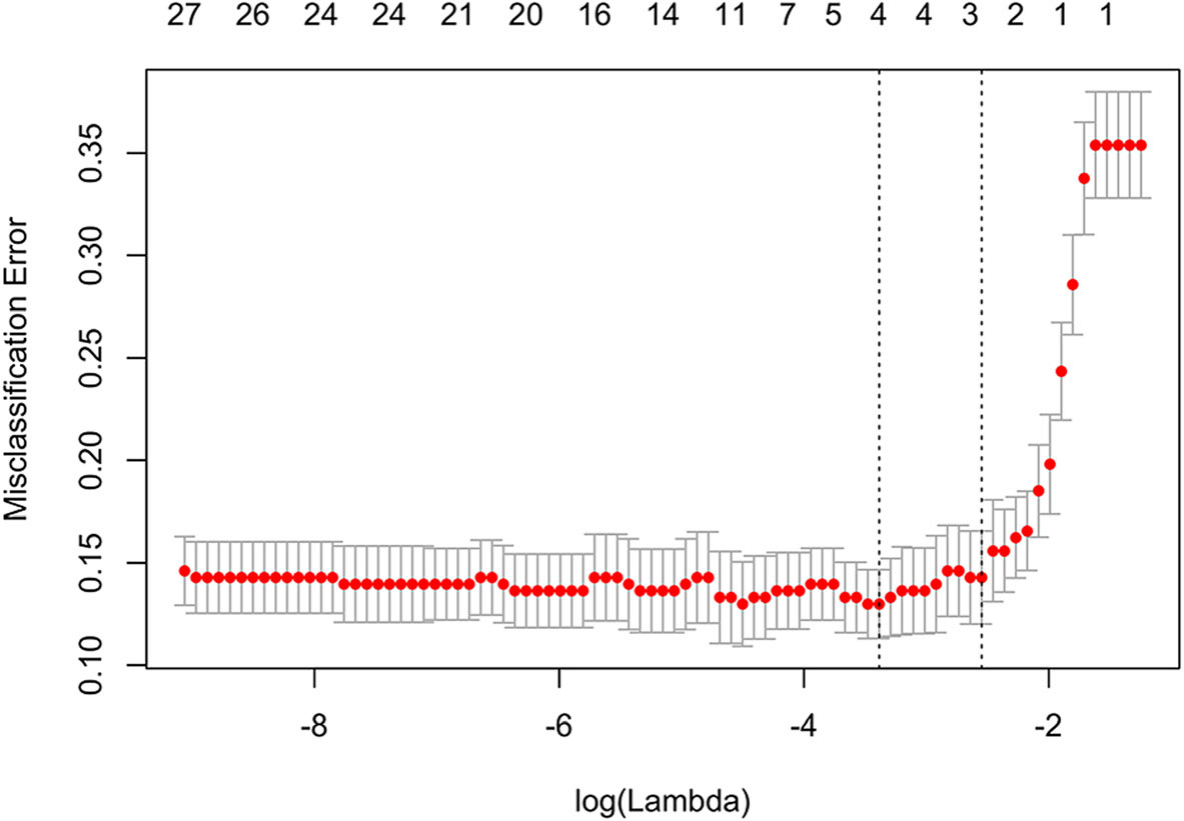
Features selection for predictive model. Tuning parameter (λ) selection in the lasso model used ten-fold cross-validation. The vertical axis shows the model misclassification rate, and the horizontal axis shows log (λ). The two vertical dashed lines represent one standard deviation on each side from the minimum value, corresponding to the chosen variables that better fit the models

The cross-correlation matrixes (Fig. 2) showed that there were multiple complex cross-correlations among the 24 parameters. Three key features (2 CT signs, 1 texture parameter) highly related with the identification of the two groups in the training set were selected with non-zero coefficients by lasso regression to establish the predictive model is depicted in Fig. 3. The 3 selected features were consequently conducted into a predictive model the Pre-scores for each patient were calculated using the calculation formula. Pre-scores = − 1.53 + 0.38*Size(cm) + 0.22*Cystoid variation-0.01*meanValue.

**Fig. 4:**
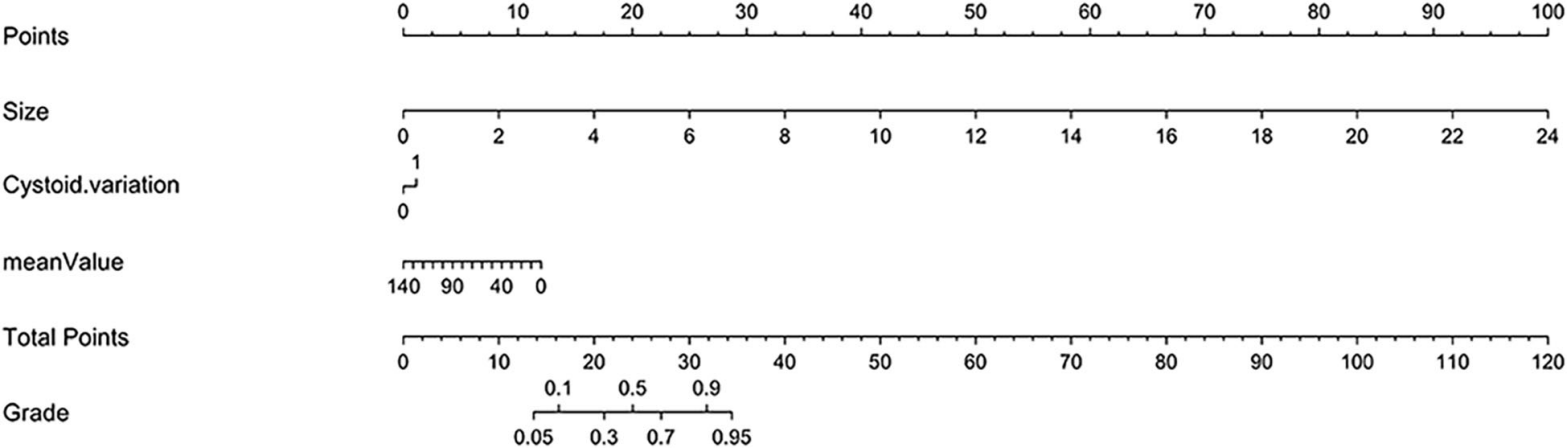
Developed prediction nomogram. The nomogram was developed in the training set, with Size, Cystoid variation and meanValue incorporated. The probability of each predictor can be converted into scores according to the first scale “Points” at the top of the nomogram. After adding up the corresponding prediction probability at the bottom of the nomogram is the malignancy of the tumor. The cutoff point of our nomogram is 0.5. The patient would be diagnosed as high-malignant potential GIST when the total prediction probability is beyond the cutoff point

Finally, the 3 selected features were incorporated into the nomogram building (Fig. 4).

### Predictive performance and validation of nomogram

GIST patients in the high-malignant potential group generally had higher Pre-scores than patients in low-malignant group in both the training and validation sets (*p* values < 0.001, respectively) (Table 3).

**Table 3:** Pre-scores of low-malignant and high-malignant potential GIST patients in training and validation sets

Characteristics	Training set		*P*-value	Validation set		*P*-value
	Low-malignant group (*n* = 109)	High-malignant group (*n* = 199)		Low-malignant group (*n* = 47)	High-malignant group (*n* = 85)	
Size (mean ± SD, cm)	3.01 ± 1.01	8.22 ± 3.99	< 0.001	3.17 ± 1.01	8.46 ± 4.44	< 0.001
Cystoid variation (%)			< 0.001			< 0.001
+	31 (28.44%)	154 (77.39%)		15 (31.91%)	67 (78.82%)	
–	78 (71.56%)	45 (22.61%)		32 (68.09%)	18 (21.18%)	
meanValue	66.07 (54.55, 88.27) ^*^	51.19 (41.45, 65.41) ^*^	< 0.001	64.52 (53.34, 86.89) ^*^	51.39 (39.91, 64.50) ^*^	< 0.001
Pre-score	−0.38 (−0.67, −0.05) ^*^	1.44 (0.65, 2.73) ^*^	< 0.001	−0.38 (−0.62, −0.01) ^*^	1.44 (0.58, 3.06) ^*^	< 0.001

Note: Size: the maximal diameter on the largest cross section of tumor; ^*^Values refer to median (interquartile range (IQR)); *P*-values were the results of univariable association analyses of each characteristic and of the two groups; *SD* standard deviation; *Pre-score* prediction score

**Fig. 5:**
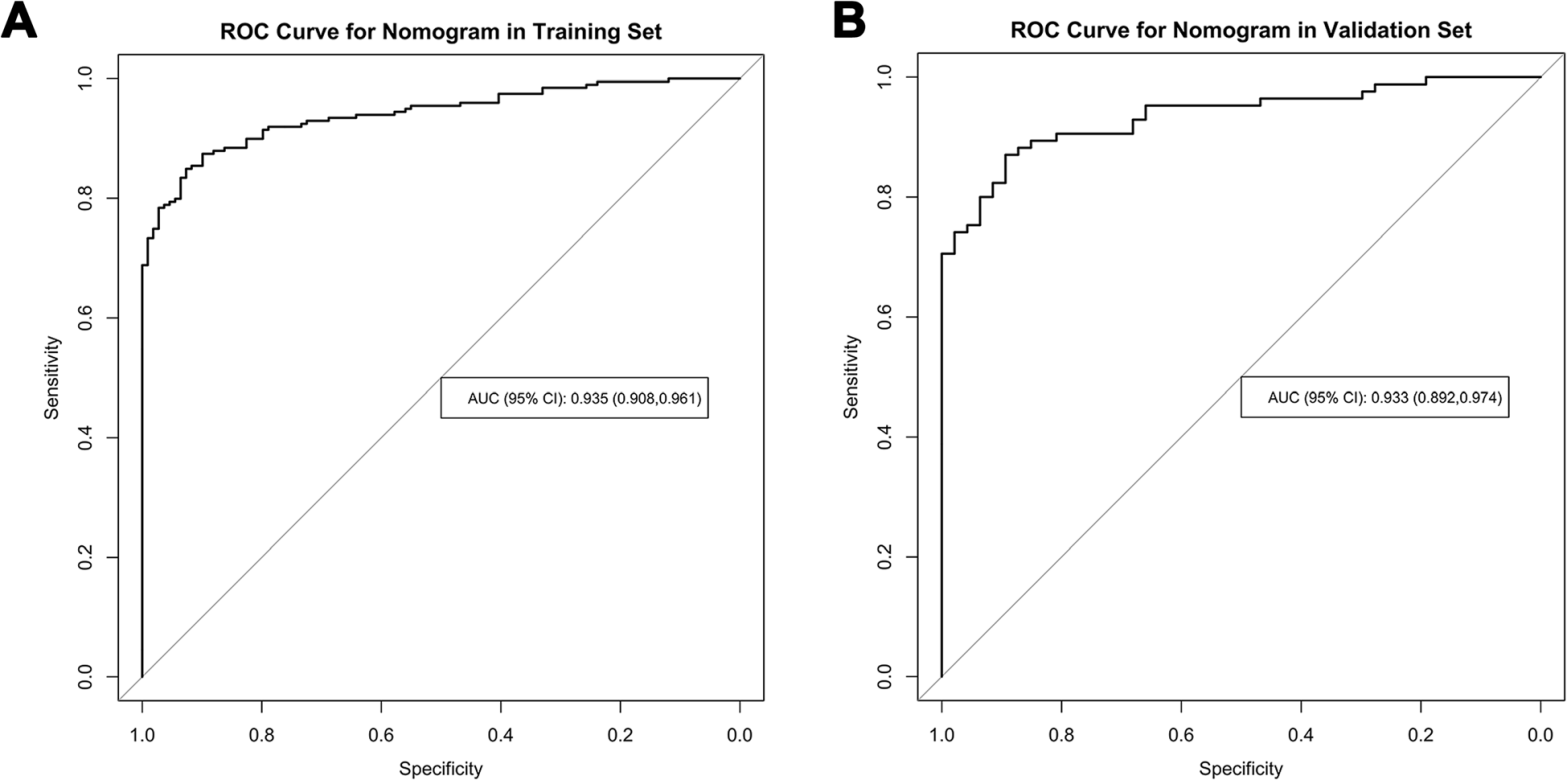
**a**, **b** Receiver-operating characteristic analysis of the prediction nomogram in training (**a**) and validation (**b**) sets

The prediction nomogram had an excellent discrimination capacity for discriminating the low- from high-malignant potential GIST in training set (AUC (95% CI) =0.935 (0.908, 0.961), sensitivity = 81.4%, specificity = 75.0%, accuracy = 88.0%) and validation set (AUC (95% CI) =0.933 (0.892, 0.974), sensitivity = 90.6%, specificity = 75.7%, accuracy = 88.6%), as shown in Fig. 5a, b.

**Fig. 6:**
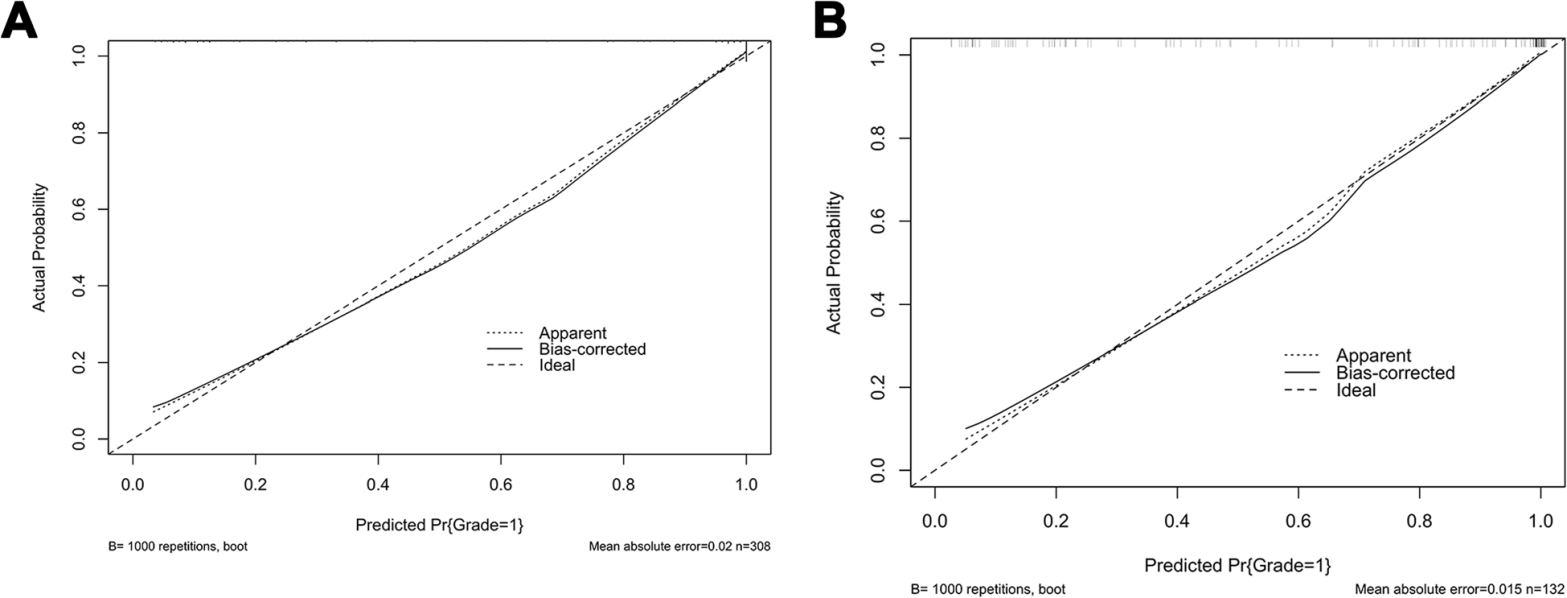
**a**, **b** Calibration curves of the prediction nomogram in training (**a**) and validation (**b**) sets. Calibration curves depict the calibration of the nomogram in terms of the agreement between the probability of the malignant potential of GISTs (Grade) and actual observation. The Y-axis represents the actual observed rates of high-malignant potential GIST whereas the X-axis represents the predicted malignancy probability estimated by the nomogram. The solid line represents the ideal reference line that predicted GIST malignant corresponds to the actual outcome, the short-dashed line represents the apparent prediction of nomogram, and the long-dashed line represents the ideal estimation. The actual GIST malignancy probability corresponded closely to the prediction of the nomogram

The calibration curve of nomogram for the probability of high-malignant potential GIST demonstrated a good agreement between prediction by nomogram and actual observation in two sets (*p* values > 0.05, respectively) (Fig. 6a, b). The C-index for the prediction nomogram was 0.941 (95% CI, 0.912 to 0.956) in the training set and 0.935 (95% CI, 0.901 to 0.982) in the validation set.

### Clinical utility of nomogram

**Fig. 7:**
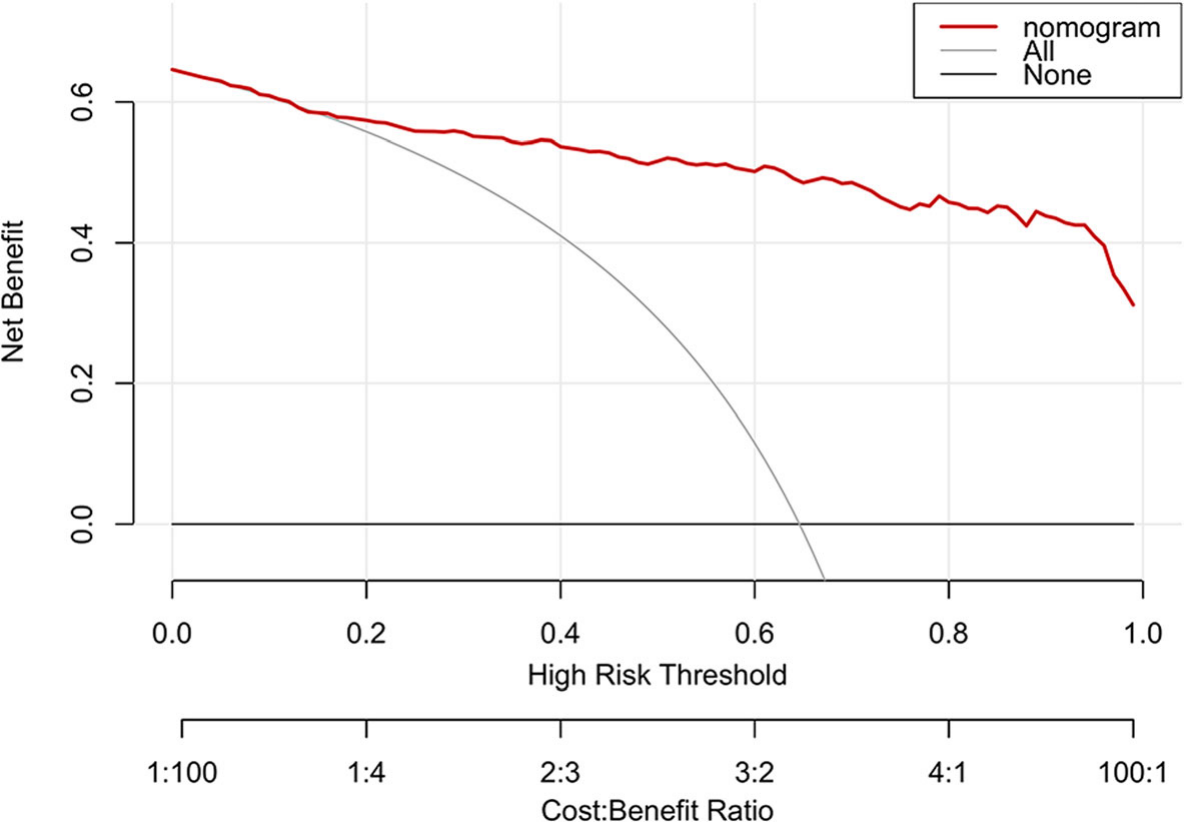
DCA for the prediction nomogram. The x-axis represented the threshold probability. The threshold probability was where the expected benefit of treatment was equal to the expected benefit of avoiding treatment. The y-axis represented the net benefit. The red line represented the prediction nomogram. The grey and black line represented the hypothesis that all patients with GIST were high-malignant potential or low-malignant potential, respectively

The DCA for the prediction nomogram was presented in Fig. 7. The decision curve showed that if the threshold probability of a patient or doctor is > 10%, using the prediction nomogram to predict GIST malignant potential would add more benefit than either the “treat all patients as low-malignant” or the “treat all patients as high-malignant”.

## Discussion

In this study, a prediction nomogram was developed and validated for the preoperative individualized prediction of the malignant potential in GIST patients. The prediction nomogram consisting of 2 CT signs (size and cystoid variation) and 1 texture parameters (meanValue), which was easily available preoperatively, successfully stratified GIST patients according to their malignant potential.

GISTs have become the model of targeted therapy for solid tumors with the in-depth study of molecular pathology, and the choice of treatment for GISTs is closely related to their risk stratification [22]. GISTs are classified into 4 risk categories determined by the tumor size, location and mitotic index of pathology [3]. Previous researches suggest that completely surgical resection is regarded as the main treatment for GIST patients at very low- and low-risk and they could be followed up regularly as benign tumors after resection, while patients with intermediate- and high-risk are required to take imatinib mesylate in addition to the operation to prevent metastasis or postoperative recurrence [23–25]. Tumors size and location are relatively easy to obtain preoperatively using anatomic imaging methods, such as positron emission tomography-computed tomography (PET/CT) and diffusion-weighted magnetic resonance imaging (MRI) besides CT, but it is difficult to preoperatively calculate the mitotic index except for invasive biopsy. Although PET/CT is the most sensitive and accurate method [26], it is not recommended as a routine examination because of the high cost and great radiation damage [27]. MRI may be another method that could provide functional quantitative indicators like ADC values, which can be used for GIST malignancy assessment, but conventional MR signs, such as the degree of GIST enhancement, are limited to predict the risk grade of GISTs before surgical resection [28]. Therefore, CT-based predictive nomogram in discriminating malignant potential of GISTs could have better generalizability and clinical application value. Previous research has shown that quantitative features extracted from CT images might be a potential imaging biomarker for mitotic count of GISTs in a noninvasive way [29].

In this study, size and cystoid variation of CT signs and meanValue of texture parameters, which were most associated with the malignant potential of GISTs, were selected to establish the prediction nomogram. Tumor size has been confirmed to be positively correlated with the malignancy of GISTs [29–31]. The maximal diameter on the largest cross section of tumor in high-malignant potential GISTs was larger than that in low-malignant GISTs in both the training and validation sets (*p* values < 0.001, respectively) (Table 3), the results of this study are consistent with the conclusion of the above reports. Similarly, some scholars believe that the presence of cystic degeneration and necrosis within the mass can be used as a reliable index to evaluate GISTs malignancy [32, 33]. This hypothesis may be related to the fact that with the increase of malignancy of tumors, cystic degeneration and necrosis are more likely to occur inside the mass when the rate of differentiation and proliferation of tumor cells far exceeds the rate of proliferation of blood vessels. In addition, low-malignant potential GIST patients usually exhibit higher meanValue than high-malignant ones. The meanValue in histogram which represent the average value of ROI reflects the degree of texture regularity: the larger the value, the more regular the texture is, that is, the lower the heterogeneity is. Heterogeneity is a recognized feature of malignant tumors and considered to be positively correlated with the malignancy of tumors, which is of great clinical significance [34, 35]; the results of this study are consistent with this correlation.

Wang C et al. developed a radiomic nomogram consisting of the maximum diameter, location of tumor and intensity values range of radiomics to differentiate the high– from the low–malignant potential GISTs (AUCs = 0.882 (training set), 0.920 (validation set), respectively) [29]. Nevertheless, the nomogram established in present study holds greater individualized prediction for GIST patients (AUCs = 0.935 (training set), 0.933 (validation set), respectively), which is more valuable for the current trend toward personalized medicine. This discrepancy may be related to the texture features extracted from the 3D spatial analysis can more accurately reflect the heterogeneity of tumor than 2D images [36]. Note that tumor location did not show enough predictive strength with malignancy in GISTs, which may be connected with the grouping criteria (gastric vs. non-gastric) in this study. The most important and final argument for the clinical use of the nomogram is based on the need to interpret individual need of additional treatment. The decision curve showed that if the threshold probability of a patient or doctor is > 10%, using the prediction nomogram to predict the malignancy of GISTs adds more benefit than either the treat-all-patients as high-malignant potential or the treat-all-patients as low-malignant potential.

However, the present study had several limitations although the results were encouraging. First, this study was a single-center retrospective study, and it is necessary to design a new multi-center study for further evaluation and verification of the results. Second, the sample selection was biased in this retrospective study, and a prospective study is required to confirm and validate the nomogram. Third, the texture features extracted in this study were based only on portal venous-phase CT images. Whether the use of other periods, such as arterial-, delayed-phase images or the combination of them will increase the predictive efficiency in the malignant potential of GISTs needs further study.

## Conclusions

A prediction nomogram based on CT and texture analysis was constructed and validated in our study, which was conveniently used to facilitate the preoperative individualized prediction of malignant potential in GIST patients.

## Supplementary information

**Additional file 1: Table S1.** Specific categories of texture parameters.

## Data Availability

Yes

## Abbreviations

3D: Three-dimensional
AUC: The area under ROC curve
C-index: Concordance index
CT: Computed tomography
DCA: Decision curve analysis
GIST: Gastrointestinal stromal tumors
GLCM: Gray-level co-occurrence matrix
ICCs: Intra- and interclass correlation coefficients
Lasso: Least absolute shrinkage and selection operator
MRI: Magnetic resonance imaging
NIH: National Institutes of Health
PACS: Picture archiving and communication system
PET/CT: Positron emission tomography-computed tomography
Pre-score: Prediction score
ROC: Receiver operating characteristic
ROI: Region of interest
