# Supplementary material for: Development and validation of a nomogram based on CT images and 3D texture analysis for preoperative prediction of the malignant potential in gastrointestinal stromal tumors
Source: Cancer Imaging. 2020 Jan 13;20:5. doi: 10.1186/s40644-019-0284-7 (PMC6958787; doi:10.1186/s40644-019-0284-7)
Supplement: Supplementary file 1 — Additional file 1: Table S1. Specific categories of texture parameters. [file 40644_2019_284_MOESM1_ESM.docx]

| **Table S1** Specific categories of texture parameters | | |
| --- | --- | --- |
| Type | Feature Name (Symbol/abbreviation) | Description |
| Histogram |  |  |
|  | Skewness (-) | Measures the asymmetry of the gray-level distribution in the histogram. |
|  | Kurtosis (-) | Measures whether the gray-level distribution is peaked or flat relative to a normal distribution. |
|  | Entropy (Entropy H) | Measures the randomness of the distribution. |
|  | Energy (Energy H) | Measures the uniformity of the distribution. |
|  | Minvalue (-) | Measures the minimum in the Volume of Interest. |
|  | Meanvalue (-) | Measures the average in the Volume of Interest. |
|  | Stdvalue (-) | Measures the standard deviation in the Volume of Interest. |
|  | Maxvalue (-) | Measures the maximum in the Volume of Interest. |
| GLCM (Gray-level co-occurrence matrix) |  |  |
|  | Homogeneity (-) | Measures the homogeneity of gray-level voxel pairs. |
|  | Energy (-) | Also called Uniformity or Second Angular Moment, measures the uniformity of gray- level voxel pairs. |
|  | Contrast (-) | Also called Variance or Inertia, measures the local variations in the GLCM. |
|  | Correlation (-) | Measures the linear dependency of gray-levels in GLCM. |
|  | Entropy (-) | Measures the randomness of gray-level voxel pairs. |
|  | Dissimilarity (-) | Measures the variation of gray-level voxel pairs. |
